# Supplementary material for: Three-dimensional single-particle reconstruction by atomic force microscopy imaging and template matching allows rapid structural-based validation of recombinant SARS-CoV-2 Spike protein from a single topology image
Source: Nanoscale Horiz. 2026 Jul 25. Online ahead of print. doi: 10.1039/d6nh00151c (PMC13418849; doi:10.1039/d6nh00151c)
Supplement: NH-OLF-D6NH00151C-s001 [file NH-OLF-D6NH00151C-s001.pdf]

Supplementary Information (SI) for Nanoscale Horizons.
This journal is © The Royal Society of Chemistry 2026

Three-dimensional single-particle reconstruction by atomic force microscopy imaging and template matching allows rapid structural-based validation of recombinant SARS-CoV-2 Spike protein from a single topology image

SUPPLEMENTARY INFORMATION

Liisa Lutter [1], David M. Beal [1], Maria Stanley [1], Joanne Roobol [1], Sarah Martin [1], James D. Budge [1], Phoebe E. Lee [1], Emi Nemoto-Smith [1], Ian Brown [1], Martin J. Warren [1,2]*, C. Mark Smales [1,3]*, Wei-Feng Xue [1]*

[1]School of Natural Sciences, University of Kent, CT2 7NJ, Canterbury, UK

[2]Quadram Institute Bioscience, Norwich Research Park, Norwich, NR4 7UQ, UK

[3]National Institute for Bioprocessing Research and Training, Foster Avenue, Mount Merrion, Blackrock, Co. Dublin, A94 X099, Ireland

* Correspondence to: C.M.Smales@kent.ac.uk, M.J.Warren@kent.ac.uk and W.F.Xue@kent.ac.uk

SUPPLEMENTARY FIGURES

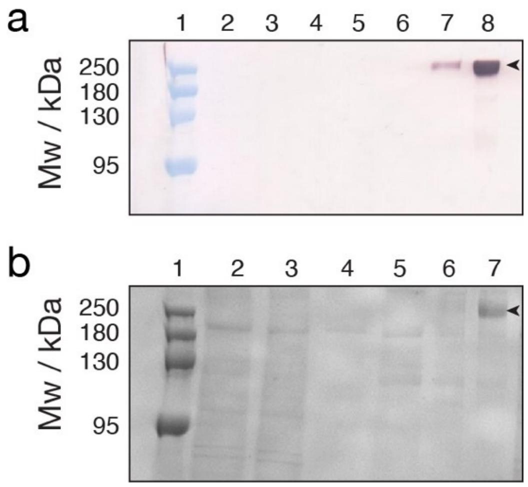

Supplementary Figure S1 *Western blot and SDS-PAGE analysis of the purified recombinant Spike protein produced and secreted from CHO-S cells. (a) Western blot analysis of the purified Spike protein. Lanes contain: 1) Molecular weight marker; 2) cell culture supernatant after dialysis; 3) flowthrough after loading column; 4) binding buffer flowthrough; 5) flowthrough from 30 mM imidazole wash buffer; 6) flowthrough from 50 mM imidazole wash buffer; 7) eluted protein in 50 mL elution buffer; 8) concentrated and buffer exchanged eluted protein. (b) Coomassie stained SDS-PAGE analysis of the purified Spike protein produced and secreted from CHO-S cells. Lanes contain: 1) Molecular weight marker; 2) cell culture supernatant after dialysis; 3) flowthrough after loading column; 4) binding buffer flowthrough; 5) flowthrough from 30 mM imidazole wash buffer; 6) flowthrough from 50 mM imidazole wash buffer; 7) concentrated and buffer exchanged eluted protein. Spike protein bands are indicated with an arrow.*

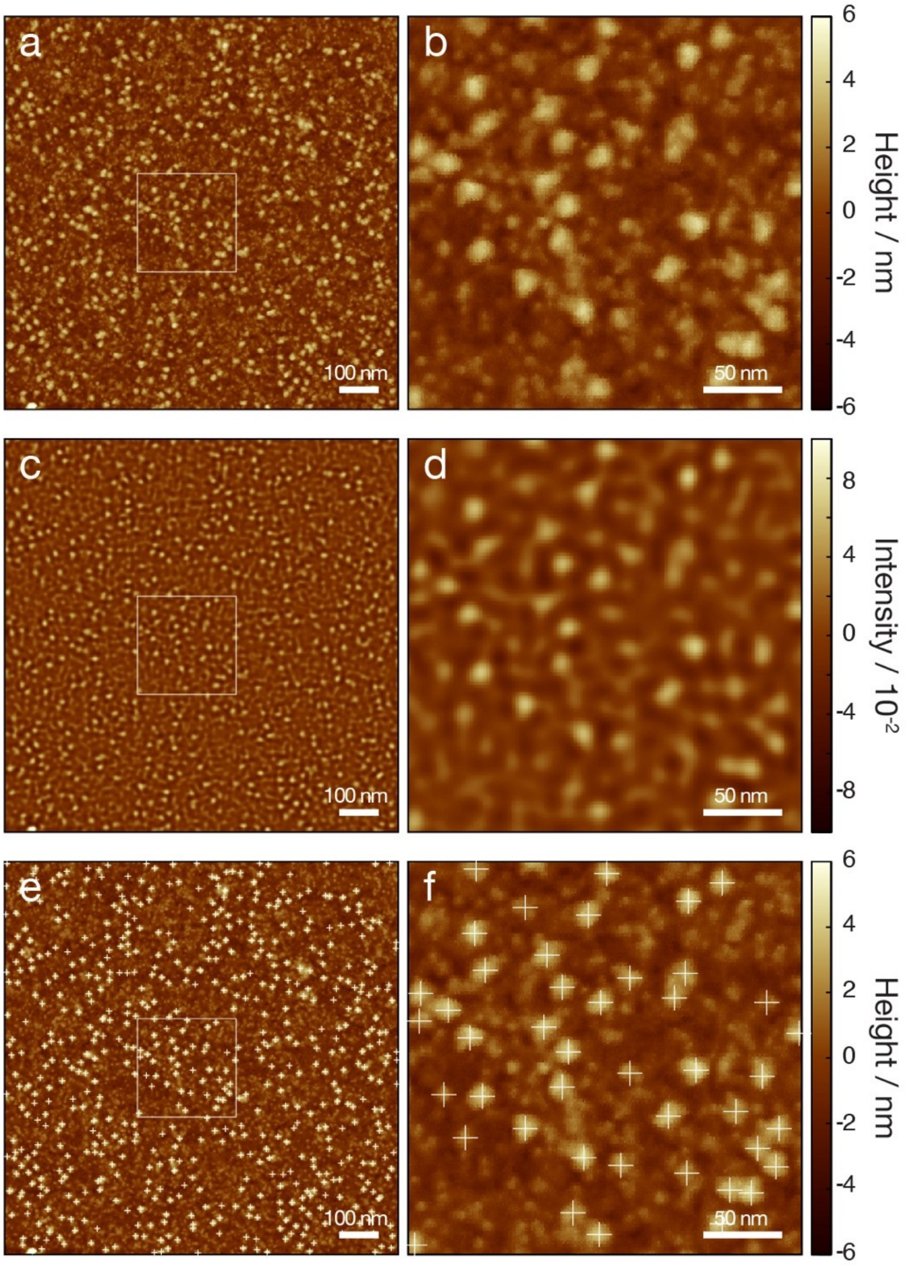

Supplementary Figure S2 *Automated and objective picking of S protein particle images by a Laplacian of Gaussian convolution image filter. (a) Representative height topographic image of a 1024x1024 pixels, 1 µm × 1 µm area scan of the specimen surface. The image is the same as shown in* ***Fig. 2a****. (b) A 4× magnified view of the area indicated by the white box in (a). (c) Laplacian of Gaussian filtered image of (a). (d) A 4× magnified view of the area indicated by*

the white box in (c). (e) The same height topology image as (a) with the x- and y-coordinates of detected particles labelled with crosses. These coordinates are subsequently used for extraction of the particle images. (f) A 4× magnified view of the area indicated by the white box in (e). The scale bars represent 100 nm in (a), (c) and (e), and 50 nm in (b), (d) and (f).

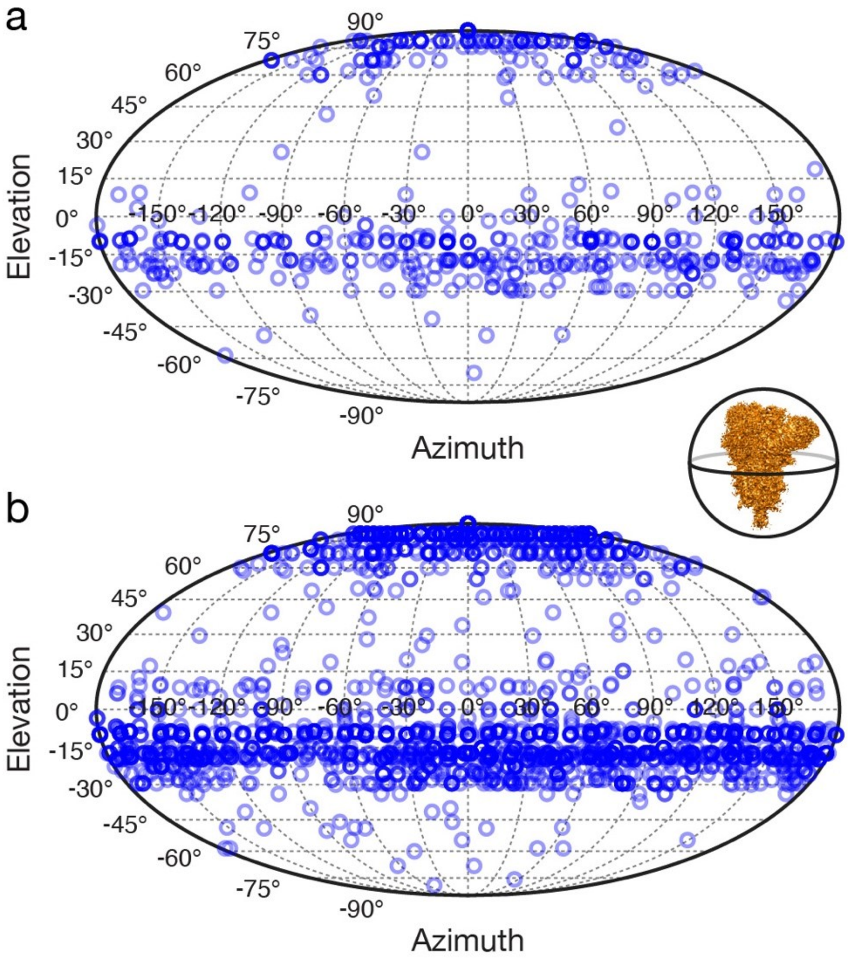

Supplementary Figure S3. *Distributions of orientations for particles picked and used in the 3D single-particle surface envelope reconstruction. (a) Azimuth and elevation angles for 582 particles picked from image shown in* ***Fig. 2a****. (b) Azimuth and elevation angles for all 2568*

particles picked from all images. Inset shows the reference orientation for the Spike protein, which is the original orientation of the EMDB map EMD-21452 of the S protein [20]. The Azimuth and elevation angles for individual particle image are show as circles projected onto an equal-area Mollweide projection of a spherical surface.

SUPPLEMENTARY MOVIE

Supplementary Movie S1. *3D contact-point cloud and sliced planes of the 3D volumetric contact point density map of the S protein 3D surface envelope reconstruction obtained from a single AFM height topology image. The 3D contact-point cloud (dots) is the same as shown in* ***Fig. 5*** *and was reconstructed from 582 particles extracted from an experimental AFM image shown in* ***Fig. 2a****. The AFM-based surface envelope density maps are shown together with the cryo-EM map EMD-21452 of the S protein [20]. The AFM-based densities along the sliced planes are represented by the colour intensity identical to* ***Fig. 5****.*
